# Knowledge Translation Improvement Strategies in Universities of Medical Sciences in Iran: A Qualitative Study

**DOI:** 10.4314/ejhs.v31i1.15

**Published:** 2021-01

**Authors:** Sogand Tourani, Narges Rafiei, Shahnaz Rimaz, Seyed Kazem Malakouti, Alireza Heidari

**Affiliations:** 1 Department of Health Services Management, School of Health Management and Information Science, Iran University of Medical Sciences, Tehran, Iran; 2 Radiation Biology Research Center, Department of Epidemiology, School of Public Health, Iran University of Medical Sciences, Tehran, Iran; 3 Mental Health Research Center, School of Behavioral Sciences and Mental Health, Iran University of Medical Sciences, Tehran, Iran; 4 Health Management and Social Development Research Center, Golestan University of Medical Sciences, Gorgan, Iran

**Keywords:** Knowledge translation, Improvement strategies, Medical universities

## Abstract

**Background:**

Recently, one of the challenges in the health system of the country is the need for research contributing to policy-making. Therefore, it is crucial to develop activities in the field of knowledge Translation (KT). This study aimed to propose KT improvement strategies in universities of medical sciences in Iran.

**Methods:**

In this qualitative study, 18 semi-structured interviews were conducted with key informants from the medical universities in Iran during January-July 2018. The transcribed documents were analyzed using the Gale framework analysis approach. Data organization was carried out using MAXQDA version 10 software.

**Results:**

According to framework analysis, six KT improvement strategies were identified including improving the abilities and skills of researchers, improving the processes and quality of knowledge production, revising policies and laws, improving the prerequisites, culture-building, and promoting the use of evidence.

**Conclusion:**

Given the challenges and strategies outlined in this study, it seems that the mechanism of KT and its effects on improving health plans for policymakers and researchers has not been elucidated yet. Therefore, considerable changes in prerequisites, knowledge production processes, academic procedures, policies and laws are necessary for implementing KT in universities of medical sciences in Iran.

## Introduction

Knowledge Translation (KT) is defined as the process of exchange, synthesis and ethically sound application of knowledge to improve health, provide more effective health services and products, and strengthen the healthcare system in a complex system of interactions between health researchers and users (1). In addition, KT includes the use of high quality knowledge in decision-making processes (2). The essential attributes of KT include the emphasis on the use of knowledge in practice and continuous participation of knowledge producers and users (3), which yield consequences such as community health promotion, provision of more effective health services, improvement in practice, strengthened healthcare system, decreased the adverse effect of care and the length of stay at hospital, and ultimately, reduced financial burden on patients, the health system and community (3,4). On the other hand, inability to translate research results into practice in healthcare leads to inequality, waste of time and time consuming studies (5,6), thereby depriving some patients of standard treatment recommendations or exposing them to harmful or unproven treatments in the field of acute or chronic care or even preventive care (7).

In 2005, the World Health Organization (WHO) emphasized the establishment or strengthening of mechanisms for knowledge transfer to support evidence-based public health and healthcare delivery systems (8).

Currently, one of the challenges in the health system is the need for research contributing to policy-making. In this regard, there is a need for the development of health system research, especially in the area of KT activities (9). Despite the considerable expansion in the last few years, native health system research is still in the first stages. Therefore, policymakers face a lack of reliable evidence in responding to some questions (10). In most cases, there is a lack of timely provision of evidence to policymakers. In addition, the evidence might not be transferred with the proper language or might not be noted by policymakers (11).

Based on the previous studies, the situation of KT in Iranian medical sciences is not appropriate (12,13), and in recent years, senior managers of the health system of the country have emphasized those policy-makings that are formed based on the best evidence and results of scientific studies, the country's status and viewpoints of key stakeholders (9). While some studies have assessed the status of KT in universities of medical sciences (12,13) and barriers of KT in Iran (14, 15), no similar qualitative study has been conducted. With this background in mind, this study aimed to propose KT improvement strategies in Iranian universities of medical sciences.

## Materials and Methods

This qualitative study was conducted to investigate the KT improvement strategies in universities of medical sciences in Iran. It is worth noting that the participants in the present study were among key informants in the field of KT that were purposefully selected from faculty members working in KT centers affiliated to the 5 medical universities. Inclusion criteria were being a faculty member with at least three years of experience and willingness to participate in the study. Data were collected thorough semistructured and in-depth interviews with participants during January-July 2018. The interview guide was prepared based on the literature review and study goals. In order to assess the validity of the interview guide, the interview questions were evaluated and corrected by the research team, and the interview guide was tested on three nonparticipants to confirm the number and order of the questions. Interviews were conducted in the interviewees' office by a trained member of the research team (NR). The interviews continued to reach saturation until no new strategy was presented by interviewees. Therefore, a total of 18 semi-structured interviews were conducted, each lasting 30–60 minutes (with a mean of 40 minutes). Three participants answered the questions in written form.

In this study, we used framework analysis method for data analysis. In this regard, Gale et al. (2013) introduced a complete model of framework analysis consisting of 7 steps (16): (1) one of the authors transcribed the recorded interviews, (2) two research team members were familiarized with and immersed in the data after listening to interviews several times and reading the text of the interviews, (3) the first three interviews were coded independently by each individual after each interview (inductive coding), also related literatures were reviewed, and the codes extracted from the literature review were collected (deductive coding) (4). At this point, all the researchers agreed on a set of codes over a two-hour session. Afterwards, codes were grouped into categories, and an analytical framework was developed to apply this coding to other interviews. The analytical framework, of course, was revised until coding the last interview (5). Subsequent interviews were coded according to agreed codes and themes (6). In the next stage, the codes were extracted in the form of categories (7). In the final step, all the initial interpretations, codes, and themes were reviewed by all members of the research team during two 60-minute sessions, and the final interpretation was performed.

For assessing the quality of the current qualitative study, we ensured four trustworthiness criteria suggested by Lincoln and Guba such as credibility, transferability, dependability and, conformability (17).

Data analysis and organization were carried out using MAXQDA version 10 software. Notably, the ethical considerations included receiving a code of ethics from the Ethics Committee of the Iran University of Medical Sciences (IR.IUMS.REC 1395.28656), attaining a written consent from the participants to record voices, ensuring the subjects of the confidentiality terms regarding their personal information, and permitting interviewees to leave the study at their request.

## Results

Interviewees are described based on participants' type of work experience and years of experience in Table 1.

**Table 1:** Characteristics of participants

Participant	Sex	Type of work experience	Experience (Year)
**1**	M	Faculty member, Member of knowledge translation center	28
**2**	M	Faculty member, Member of knowledge translation center	21
**3**	F	Faculty member, Member of knowledge translation center	30
**4**	M	Faculty member, Research deputy	21
**5**	F	Faculty member, Member of knowledge translation center	23
**6**	F	Faculty member, Research manager	21
**7**	F	Faculty member, Member of knowledge translation center	10
**8**	F	Faculty member, Member of knowledge translation center	24
**9**	M	Faculty member, Member of knowledge translation center	10
**10**	F	Faculty member, Member of knowledge translation center	26
**11**	F	Faculty member, Member of knowledge translation center	14
**12**	F	Faculty member, Member of knowledge translation center	16
**13**	M	Faculty member, Member of knowledge translation center	18
**14**	M	Faculty member, Member of knowledge translation center	22
**15**	M	Faculty member, Member of knowledge translation center	23
**16**	M	Faculty member, Member of knowledge translation center	17
**17**	F	Faculty member, Member of knowledge translation center	12
**18**	F	Faculty member, Member of knowledge translation center	16

Six themes and 34 sub-themes were identified based on the framework analysis, including improving the abilities and skills of researchers (4 sub-themes), improving the processes and quality of knowledge production (4 sub-themes), revising policies and laws (4 sub-themes), culture-building (4 sub-themes), improving the prerequisites (13 sub-themes) and promoting the use of evidence (5 sub-themes) (Table 2).

**Table 2:** Identified themes and sub-themes for KT improvement strategies

Theme	Sub-theme
**Improving the** **abilities and skills of** **researchers**	Introducing and enhancing researchers' knowledge of KT, idea registration and patenting Empowering researchers in the field of commercialization Selection of research line by any researcher Strengthening teamwork skills (collaboration, interaction, and participation) between researchers together and between researchers with knowledge users
**Improving knowledge** **production processes** **and quality** **(production of** **applied knowledge** **based on stakeholder** **needs)**	Researchers' cooperation with knowledge users in selecting research topics and priorities as well as in interpreting and applying findings Production of applied knowledge based on stakeholders needs Providing research results in the language of the stakeholders (Persian language and comprehensible literature) Accurate supervision on research process and establishing a quality assurance system
**Revising policies and** **laws**	Revising faculty members' tenure and promotion criteria such as article publishing and creating incentive mechanisms and putting value into doing applied and problem-solving research Institutionalizing sustainable policies based on KT and application of knowledge Developing comprehensive patent registration laws and protecting intellectual property rights and promoting legal frameworks for the use of clinical trial results Changing the university's goal from education to addressing community problems and prioritizing KT for the education system
**Culture-building**	Valuation for knowledge sharing and creation of knowledge networks Strengthening the new academic system Extensive advertisement for KT at seminars and conferences Reforming researchers' attitudes toward publishing books and articles to conduct applied research and need-based work
**Improving the** **prerequisites**	Funding for applied research Reducing hierarchy and facilitating the process of doing applied research Facilitating the processes of attracting research order from stakeholders Strengthening the “Industrial Relations Office” at the university and receiving research funding from audiences and industry Defining the process of using the existing data in the health and industry section by university researchers Encouraging cross-sectoral projects and defining their processes Facilitating communication between researchers and universities inside and abroad Applying good and efficient management forces Reducing rotation and change of university research and executive managers Changing the attitude of researchers and managers toward KT Strengthening and updating of internal databases and creating an integrated information system from completed and ongoing research at the university Training knowledge brokers Establishing an R&D unit at the university
**Promoting the use of** **evidence**	Improving the knowledge of users about the university's activities through conferences, joint meetings, workshops, website, and provision of policy brief Upgrading knowledge absorptive capacity and its use in knowledge recipients Applying the proper techniques to transfer research results to stakeholders Gaining confidence in the consumers of science Assigning incentives to knowledge users to use research results

**Improving the researchers' abilities and skills**: While the concept of KT has been introduced in the country for more than a decade, many researchers and managers are still unaware of this issue. Therefore, one of the strategies mostly proposed by the majority of interviewees was familiarizing researchers with the concept of KT. In addition, most interviewees believed that while researchers in Iran are skillful knowers, they are not talented transmitters, users, or even listeners. A researcher must have features and capabilities to guide knowledge toward ability. In this context, enabling researchers in the field of commercialization was one of the proposed strategies:

“*A researcher must have the necessary skills of a knowledge broker. It means that in addition to the research conducted, the researcher is expected to get up from behind the desk and come to the industry. This person must know the language of industry and be able to attract society, advertise and be familiarized with marketing. In addition, the researcher must know the negotiation techniques and how to conclude contracts*”. *[Interviewee 3]*

**Improving knowledge production processes and quality**: Researchers' collaboration with knowledge users in selecting research topics and priorities, as well as in interpreting and applying the results, were strategies emphasized by most interviewees. One of the main weaknesses of KT in Iran is the separation of researchers from knowledge users. In addition, since the majority of articles are published in English, they are unusable for knowledge users, such as healthcare personnel (e.g., nurses, midwives and managers). Therefore, more attention must be paid to the transmission of research results in the language of users.

In addition, the professors believed that the establishment of a quality assurance system in the university would result in welcoming the products of the university by knowledge users:

*“We must establish a quality assurance system in the university. We have to first guarantee the quality of the university's products of faculty members. Then, everyone will come to the university. However, some organizations have had very bad experiences at the university and will cooperate with them ever again”. [Interviewee 2].*

**Revising policies and laws**: “Revising faculty members' tenure and promotion criteria such as paper publishing and putting value into doing applied research” was another strategy greatly emphasized by all interviewees, which has made researchers just think about writing an article and upgrading their own rank:

*“For instance, a professor has published 19 articles in two-three months. However, how much of it is applicable? None! He knows it himself. When you ask him why, he says that because I have to have that many articles to increase my ranking! He is just worried about increasing his H-index in some ways.” [Interviewee 1]*

*“The assessment criterion for the faculty members is conducting and publishing research. Whether the result of their research helps to solve the country's problems or not, is not their concern. In other words, a faculty member with a high publication rank is more respected in society, compared to one who has attempted to solve a problem in a deprived region.” [Interviewee 5]*

The interviewees believed that the “institutionalization of sustainable policies based on KT” has an overall impact on factors affecting the process of KT promotion and improvement in the country. Since the policies of ministries and universities are usually formulated based on the 5-years Development Plan, the existence of such policies encourages both researchers and users of knowledge to work together and facilitate related processes.

Given that, in Iran, there is usually little ethical and legal commitment to using others' ideas and knowledge generated by citing its reference, which eliminates the necessary foundations for creating trust among researchers, “defining comprehensive regulations on patent registration, and support of intellectual property rights” was another strategy proposed by the interviewees.

One of the reasons for lack of referral of knowledge users to universities and lack of using universities' products is that universities do not prioritize solving problems in society and only aim at educating students. In this regard, a strategy offered was “changing the objective of universities from education to solving problems in society”:

“*In reality, the university's goal is not education, but rather solving problems in society. In other words, a university is a place for identifying problems and proposing solutions.*” *[Interviewee 2]*

**Culture building**: According to many interviewees, reforming researchers' attitudes toward publishing books and articles to perform applied and need-based research were other strategies offered to enhance KT:

“*The attitude, type of thinking and ideology of the researcher himself is very important. For many years, their goal was only to publish articles, and this approach has been deeply rooted in them.*” *[Interviewee 5]*

Given the unfavorable interaction between university and the public and lack of informing the people of the science produced in the university, one of the strategies was “strengthening the new academic system”:

“*We must strengthen the new academic system. There should not be a gap between the university and society in the new academic system. Universities belong to people, and we must allow the people to freely share their issues in universities, attend roundtables at universities and interact with students*.” *[Interviewee 2]*

While the concept of KT has been seriously introduced to medical universities in Iran, many researchers are still unaware of this phenomenon. Therefore, it was recommended that KT be extensively advertised, especially in seminars and conferences.

**Improving the prerequisites**: According to the interviewees, conducting applied and need-based research requires more funding, which is the main reason for the lack of desire of most researchers to work in this area. Therefore, allocating budgets for applied research was one of the strategies referred to by the researchers. In addition, most professors were dissatisfied with the length of the approval process and the application of the results to the users of knowledge, considering it a barrier to the interaction between researchers and knowledge users, which ultimately discourages knowledge users. Therefore, “reducing hierarchy and facilitating the process of doing applied research” was introduced as one of the main reforms:

“*An article has proposed that KT must be more efficiently occur in Iran due to the existence of a ministry called the Ministry of Health and Medical Education (MoHME). Therefore, it is not difficult to introduce knowledge to the treatment field since the hospitals and universities are in the same ministry. Meanwhile, no such thing has happened.*” *[Interviewee 6]*

Strengthening the “Group of Industrial Relations Office” at the university and facilitating the processes of attracting research orders from knowledge users and defining the processes of using the existing data in the health, and industry section were among the strategies pointed out by most interviewees. In addition, once the university receives its research funding from the industry, it will motivate researchers to attract audiences and industry to meet their needs.

In our country, many policies rely on individuals, and change of managers might result in the elimination or adding of some processes, which ultimately causes changes in university instructions, thereby increasing the duration of the project approval process. Therefore, “using good and efficient management forces and reducing the rotation and change of university research and administrative managers” was proposed by some of the professors.

According to interviewees, the positive attitude of university managers toward KT has a considerable effect on encouraging researchers, faculty members and students to carry out need-based research.

According to some of the professors, one of the main infrastructures toward the improvement of research quality, preventing repetitive work and encouraging knowledge users to use research results is “strengthening and updating internal databases and creating an integrated information system”:

“*We need information custodian in our country. There is not custodian to determine where and when to keep the information. This is mainly because we have scattered information. Have you ever worked with PubMed? Do you know when it was developed? 1879! It has not been stopped for even a day. Now, any database in Iran is full of problems*.” *[Interviewee 1]*

Given that most university researchers are not trained to perform brokers activities, and since they have limited time for such activities, it is essential to train knowledge brokers who are familiar with both the language of science and industry and can link the university and industry:

“*We need brokers since they have industry, society, marketing, and customer attraction perspectives and are familiarized with the negotiation and customer attraction techniques.*” *[Interviewee 3]*

**Promoting the use of evidence**: One of the problems reported was that knowledge users were unaware of university activities and that the capacity of knowledge absorption by the community was not as large as the knowledge produced. Therefore, there was a discrepancy in this regard, and the audiences must be empowered in terms of optimal use of the produced knowledge. In this context, some of the strategies presented include holding conferences, joint meetings, and workshops; developing websites and preparing policy brief. Mostly, university researchers publish their research results in journals or present them in conferences that are scientifically appropriate for other researchers. Therefore, “using proper methods to transfer research results to stakeholders” was one of the strategies confirmed by the majority of interviewees.

In addition, it was suggested that incentives be provided to knowledge users regarding the application of research results to encourage these people to use the research results of researchers. However, this issue needs the cooperation of the health sector or industry to provide facilities to knowledge users with the help of each other.

## Discussion

According to the participants' opinion, the overall status of KT medical universities in Iran is not suitable, and multilateral strategies must be carried out at the university and country level. Other studies performed on universities of medical sciences in the country have shown that despite the production of knowledge and an increased number of articles in Iran, there is an unfavorable KT status (13, 18). It seems that the Iranian researchers have mainly focused on knowledge production and have shown inefficient performance in other domains such as knowledge transfer and promoting the use of evidence.

One of the challenges was related to involving research result users in designing the research question. Therefore, regular meetings with target audiences are essential to designing research questions because the two-way interactions lead to familiarity with the needs of the audience and create a common culture and language in the field of research (19).

Based on the participants' opinion, most researchers only attempt to produce research articles and pay no attention to the dissemination of results for the public and policymakers. There is ample evidence that knowledge transfer alone is rarely effective, and there must be changes in the investment, implementation, and evaluation of research (20). For instance, universities must receive their research budget from the industry or the audience, in which case, the researcher will have to turn to the industry for funding.

Active presence of researchers in decisions of executive organizations and sending research messages to decision-makers and policymakers is another challenge. Therefore, given the weaknesses observed in the interaction between researchers and users of research results, a number of strategies are proposed. Most interventions proposed to strengthen KT in developed countries mainly focus on the facilitation and increase of interaction between researchers and target groups (21–23). Of course, one of the strategies that have received a lot of attention has been the revision of the faculty members' tenure and promotion criteria, as the correction of the criteria would encourage the design and implementation of community-based research with the participation of knowledge users. In addition, one of the main challenges in the field of evidence-based decision-making is the inability to use the existing data at the MoHME, which requires the design of systematic access mechanisms in the form of guidelines and organizational structures for managing data access (24).

Another strategy proposed was correcting the attitude of university research and executive managers toward KT. Factors such as lack of support of managers and desire to conduct scientific studies turn the academic environment into an unsuitable setting for use of research evidence (25, 26).

According to interviewees, most researchers deal with a shortage of time and improper communication skills to attract knowledge users. In this regard, a strategy would be training knowledge brokers in addition to holding educational classes for researchers. Generally, knowledge brokers facilitate the identification, access, assessment, interpretation, and translation of research results into policy and practice by creating a link between researchers and knowledge users (27–30).

One of the strategies emphasized by most interviewees was increasing budgets for research projects. However, it is notable that the increase in budget will not result in an improvement in KT alone. In most countries, budgets are allocated to research centers based on their assessment. Moreover, it is used as a managerial tool to monitor research centers (31).

Given the challenges and strategies outlined in this study, it seems that the mechanism of KT and its effects on improving health plans for policymakers and researchers has not been elucidated yet.

Many of the strategies proposed in this study are nationwide and the revision of a strategy alone cannot achieve the goal of KT. Therefore, considerable changes in prerequisites, knowledge production processes, academic procedures, policies and laws are necessary for implementing KT in Iranian universities of medical sciences.

One of the limitations of this qualitative study was the number of samples included in because of the limitation of access. On the other hand, one of the strengths of the present study was the opportunity of interviewing with key informants and experts working in knowledge translation centers affiliated to the 5 medical universities in Iran. So this study can inform policymakers to adopt appropriate policy for designing and implementing the KT improvement strategies in Iran.

According to the results of the current study, there are significant weaknesses in the different domains of KT. Therefore, some strategies were proposed including improving the abilities and skills of researchers, improving the knowledge production quality and processes, revising policies and laws, culture-building, improving the prerequisites, and promoting the use of evidence.

## References

[1] Tetroe J (2007). Knowledge translation at the Canadian Institutes of Health Research: a primer. Focus Technical Brief.

[2] Straus SE, Tetroe J, Graham I (2009). Defining knowledge translation. Canadian Med Associat J.

[3] Rafii F, Parvizy S, Mehrdad N, Peyrovi H, Khoddam H (2012). Clarification of knowledge translation in health system. Iran J Nurs Reaserch.

[4] Khoddam H, Mehrdad N, Peyrovi H, Kitson AL, Schultz TJ, Athlin AM (2014). Knowledge translation in health care: a concept analysis. Med J Islam Repub Iran.

[5] Graham ID, Logan J, Harrison MB (2006). Lost in knowledge translation: time for a map?. J Continuing Education Health Professions.

[6] (2004). World report on knowledge for better health.

[7] McGlynn EA, Asch SM, Adams J (2003). The quality of health care delivered to adults in the United States. New England J Med.

[8] (2004). The 58^th^ meeting of the World Health Assembly passed the following resolution.

[9] Rashidian A (2014). Policy making challenges, and the need for introducing formal structures for evidence informed decision making in the health system. Hakim Res J.

[10] Yousefi-Nooraie R, Rashidian A, Nedjat S (2009). Promoting development and use of systematic reviews in a developing country. J Evaluation Clinical Practice.

[11] Majdzadeh R, Nedjat S, Denis J, Yazdizadeh B, Gholami J (2010). ‘Linking research to action’ in Iran: Two decades after integration of the Health Ministry and the medical universities. Public Health.

[12] Gholami J, Majdzadeh R, Nedjat S (2011). How should we assess knowledge translation in research organizations; designing a knowledge translation self-assessment tool for research institutes (SATORI). Health Res Policy Systems.

[13] Gholami J, Ahghari S, Motevalian A (2013). Knowledge translation in Iranian universities: need for serious interventions. Health Res Policy Syst.

[14] Nedjat S, Gholami J, Yazdizadeh B, Nedjat S, Maleki K, Majdzadeh R (2014). Research's Practice and Barriers of Knowledge Translation in Iran. Iranian J Publ Health.

[15] Yazdizadeh B, Nedjat S, Gholami J (2009). Utilization of research in health system decision making. Hakim Res J.

[16] Gale NK, Heath G, Cameron E, Rashid S, Redwood S (2013). Using the framework method for the analysis of qualitative data in multidisciplinary health research. BMC Med Res Methodol.

[17] Polit DF, Beck CT (2014). Essentials of nursing research: appraising evidence for nursing practice.

[18] Majdzadeh R, Nedjat S, Fotouhi A, Malekafzali H (2009). Iran's approach to knowledge translation. Iranian J Publ Health.

[19] Wensing M, Grol R (2019). Knowledge translation in health: how implementation science could contribute more. BMC Medicine.

[20] Bowen S, Graham I (2013). From Knowledge Translation to Engaged Scholarship: Promoting Research Relevance and Utilization. Arch Physical Med Rehabilitation.

[21] Lavis J, Guindon G, Cameron D (2010). Bridging the gaps among research, policy and practice in ten low- and middle-income countries: Survey of researchers. Canadian Med Associat J.

[22] (2009). Knowledge Translation Strategy 2004–2009.

[23] Dobbins M, Traynor RL, Workentine S, Yousefi-Nooraie R, Yost J (2018). Impact of an organization-wide knowledge translation strategy to support evidence-informed public health decision making. BMC Public Health.

[24] Etemad K, Heidari A, Panahi MH, Lotfi M, Fallah F, Sadeghi S (2017). Challenges of access to data of Ministry of Health from the perspective of policy-makers, producers, and consumers of data: A qualitative study. Iran J Epidemiol.

[25] Salsali M, Mehrdad N (2009). Iranian nurses' constraint for research utilization. BMC Nursing.

[26] Dobbins M, Greco L, Yost J, Traynor R, Decorby-Watson K, Yousefi-Nooraie R (2019). A description of a tailored knowledge translation intervention delivered by knowledge brokers within public health departments in Canada. Health Res Policy Syst.

[27] Dobbins M, Robeson P, Ciliska D (2009). A description of a knowledge broker role implemented as part of a randomized controlled trial evaluating three knowledge translation strategies. Implementation Science.

[28] Thompson MR, Schwartz Barcott D (2019). The role of the nurse scientist as a knowledge broker. J Nursing Scholarship.

[29] Mallidou AA, Atherton P, Chan L, Frisch N, Glegg S, Scarrow G (2018). Core knowledge translation competencies: a scoping review. BMC Health Serv Res.

[30] Edwards A, Zweigenthal V, Olivier J (2019). Evidence map of knowledge translation strategies, outcomes, facilitators and barriers in African health systems. Health Res Policy and Syst.

[31] Geuna AM, Ben R (2004). University Research Evaluation and Funding: An International Comparison. Minerva.

